# Comparison of the effectiveness of awake-prone positioning and high-flow nasal oxygen in patients with COVID-19-related acute respiratory failure between different waves

**DOI:** 10.62675/2965-2774.20240065-en

**Published:** 2024-10-09

**Authors:** Mariano Esperatti, Matías Olmos, Marina Busico, Adrian Gallardo, Alejandra Vitali, Jorgelina Quintana, Hiromi Kakisu, Bruno Leonel Ferreyro, Nora Angélica Fuentes, Argentine Collaborative Group on High Flow and Prone Positioning, Javier Osatnik, Santiago Nicolas Saavedra, Agustin Matarrese, Greta Dennise Rebaza Niquin, Elizabeth Gisele Wasinger, Giuliana Mast, Facundo Juan Andrada, Ana Inés Lagazio, Nahuel Esteban Romano, Marisol Mariela Laiz, Jose Garcia Urrutia, Mariela Adriana Mogaadouro, Micaela Ruiz Seifert, Emilce Mastroberti, Claudia Navarro Moreno, Anabel Miranda Tirado, María Constanza Viñas, Juan Manuel Pintos, Maria Eugenia Gonzalez, Maite Mateos, Verónica Barbaresi, Ana Elizabeth Grimbeek, Leonel Stein, Ariel Juan Latronico, Silvia Laura Menéndez, Alejandra Dominga Basualdo, Romina Castrillo

**Affiliations:** 1 Universidad Nacional de Mar del Plata Hospital Privado de Comunidad Intensive Care Department Mar del Plata Argentina Intensive Care Department, Hospital Privado de Comunidad, Escuela Superior de Medicina, Universidad Nacional de Mar del Plata - Mar del Plata, Argentina.; 2 Swiss Medical Group Clínica Olivos Intensive Care Unit Buenos Aires Argentina Intensive Care Unit, Clínica Olivos, Swiss Medical Group - Buenos Aires, Argentina.; 3 Sanatorio Clínica Modelo de Morón Intensive Care Unit Buenos Aires Argentina Intensive Care Unit, Sanatorio Clínica Modelo de Morón - Buenos Aires, Argentina.; 4 Sanatorio de la Trinidad Palermo Intensive Care Unit Buenos Aires Argentina Intensive Care Unit, Sanatorio de la Trinidad Palermo - Buenos Aires, Argentina.; 5 University of Toronto Interdepartmental Division of Critical Care Medicine Toronto Canada Interdepartmental Division of Critical Care Medicine, University of Toronto - Toronto, Canada.; 6 Intensive Care Unit Hospital Alemão Buenos Aires Argentina Intensive Care Unit, Hospital Alemão - Buenos Aires, Argentina.; 7 Intensive Care Unit Hospital Universitario Austral Buenos Aires Argentina Intensive Care Unit, Hospital Universitario Austral - Buenos Aires, Argentina.; 8 Intensive Care Unit Clínica Olivos Buenos Aires Argentina Intensive Care Unit, Clínica Olivos - Buenos Aires, Argentina.; 9 Intensive Care Unit Sanatorio de la Trinidad Palermo Buenos Aires Argentina Intensive Care Unit, Sanatorio de la Trinidad Palermo - Buenos Aires, Argentina.; 10 Intensive Care Department Hospital Privado de Comunidad Mar del Plata Argentina Intensive Care Department, Hospital Privado de Comunidad - Mar del Plata, Argentina.

**Keywords:** Respiratory insufficiency, COVID-19, Coronavirus infections, SARS-CoV-2, Intubation, endotracheal, Hospital mortality, Prone position, Oxygen

## Abstract

**Objective:**

To compare the effectiveness of the awake-prone position on relevant clinical outcomes in patients with COVID-19-related acute respiratory failure requiring high-flow nasal oxygen between different waves in Argentina.

**Methods:**

This multicenter, prospective cohort study included adult patients with COVID-19-related acute respiratory failure requiring high-flow nasal oxygen. The main exposure position was the awake-prone position (≥ 6 hours/day) compared to the non-prone position. The primary outcome was endotracheal intubation, and the secondary outcome was in-hospital mortality. The inverse probability weighting–propensity score was used to adjust the conditional probability of treatment assignment. We then adjusted for contextual variables that varied over time and compared the effectiveness between the first and second waves.

**Results:**

A total of 728 patients were included: 360 during the first wave and 368 during the second wave, of whom 195 (54%) and 227 (62%) remained awake-prone for a median (p25 - 75) of 12 (10 - 16) and 14 (8 - 17) hours/day, respectively (Awake-Prone Position Group). The ORs (95%CIs) for endotracheal intubation in the Awake-Prone Position Group were 0.25 (0.13 - 0.46) and 0.19 (0.09 - 0.31) for the first and second waves, respectively (p = 0.41 for comparison between waves). The ORs for in-hospital mortality in the awake-prone position were 0.35 (0.17 - 0.65) and 0.22 (0.12 - 0.43), respectively (p = 0.44 for comparison between waves).

**Conclusion:**

The awake-prone position was associated with a reduction in the risk of endotracheal intubation and in-hospital mortality. These effects were independent of the context in which the intervention was applied, and no differences were observed between the different waves.

## INTRODUCTION

A subset of patients with coronavirus disease 2019 (COVID-19) develop acute respiratory failure and acute respiratory distress syndrome (ARDS) and require invasive mechanical ventilation.^(1)^ This condition is associated with mortality rates exceeding 40% in both developed and developing countries.^(2)^ Awake-prone position (AW-PP) has been shown to reduce the risk of endotracheal intubation (ETI) in patients with COVID-19-related acute respiratory failure (ARF) receiving noninvasive advanced respiratory support in the intensive care unit (ICU) setting.^(3,4)^ The evidence showing the efficacy of AW-PP comes from studies conducted mainly during the early stage of the pandemic.^(4)^ Many aspects changed during the course of the pandemic. The incidence and rate of hospitalizations varied, challenging health systems to address different levels of stress.^(5,6)^ Vaccination coverage has also changed over time in different health systems.^(7,8)^ Similarly, severe acute respiratory syndrome coronavirus 2 (SARS-CoV-2) presents multiple variations in its genetic sequence, leading to the development of new waves of COVID-19.^(9-11)^ Even though these variables may influence the clinical outcome of individual COVID-19 patients, treatments that have proven to be effective have not been evaluated for effectiveness throughout the pandemic, nor have their effects been compared at different times during its course.

In the present study, we aimed to compare the effectiveness of the awake-prone position on relevant clinical outcomes in patients with COVID-19-related acute respiratory failure requiring high-flow nasal oxygen between different waves in Argentina. We hypothesized that the treatment effect could vary over time due to the multiple variables that changed throughout its course.

## METHODS

We conducted a prospective, multicenter cohort study at 6 ICUs at 6 centers in Argentina. The study was registered at ClinicalTrials.gov (NCT05178212) and reported following guidelines from STROBE (Supplementary Material).^(12)^ The internal review boards of the 6 centers approved the study, and informed consent was waived (Supplementary Material). The interventions carried out were part of the usual practice at each center, and the researchers guaranteed the confidentiality of their patients’ information. A detailed description of the methods, procedures related to the evaluated intervention, and statistical analysis have been previously reported (Supplementary Material).^(13)^

### Course of the pandemic and context of the study (Supplementary Material)

**Figure 1:**
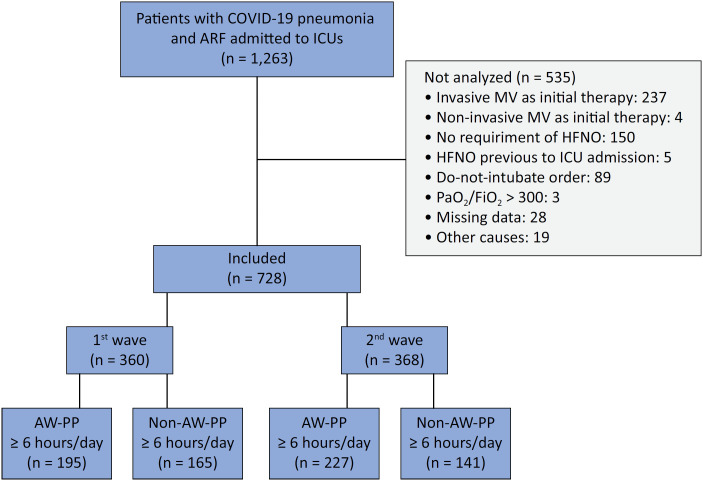
Patient inclusion.

The study period covered from March 2020 to September 2021. The first wave was considered to run from February 27, 2020, to February 16, 2021, with patients included after this wave corresponding to the second wave.^(14)^ The predominant variants identified during this period are shown in figure 1S (Supplementary Material).

### Study population

We consecutively included patients aged 18 years and older who were admitted to the ICU with a confirmed diagnosis of COVID-19 and who had been receiving high-flow nasal oxygen (HFNO) for at least 4 hours. Patients received HFNO when any of the following criteria were present: peripheral oxygen saturation (SpO_2_) < 92% with oxygen > 4L/minute; increased work of breathing with the use of accessory respiratory muscles and a respiratory rate > 30/minute; and partial pressure of oxygen/fraction of inspired oxygen (PaO_2_/FiO_2_) ratio < 200mmHg. Patients with a decreased level of consciousness, presence of shock, immediate need for intubation,^(15)^ use of positive-pressure ventilation prior to HFNO, or do-not intubate order were excluded.

### Study procedure (Supplementary Material)

Immediately after admission to the ICU, the eligibility criteria were assessed, and HFNO was started at a flow between 50 and 70L/minute, with the minimal FiO_2_ necessary to obtain an SpO_2_ > 92%. Patients who tolerated HFNO for the following 4 hours were included. The health care team encouraged and assisted all the participants in rotating from the supine position to the prone position for as long as possible, taking breaks for personal hygiene and eating. When prone positioning was not tolerated by patients, they were assisted to remain in the lateral position, alternating right and left decubitus for as long as they could tolerate it. The supine position was allowed where patients could not tolerate any of the positions mentioned. These interventions were maintained during the study period until one of the following criteria was met: maintenance of SpO_2_ > 92% with FiO_2_ ≤ 40%, flow ≤ 40L/minute for a period > 12 hours in the supine position, or ETI. Once clinical and gas stability were obtained with HFNO, the flow and oxygen were progressively weaned on a protocolized basis (Supplementary Material).^(13)^ Analgesic drugs (opioids, paracetamol) or light sedation (dexmedetomidine) were allowed and indicated according to the criteria of the health care team (Supplementary Material).

### Variables and measurements

**Figure 2:**
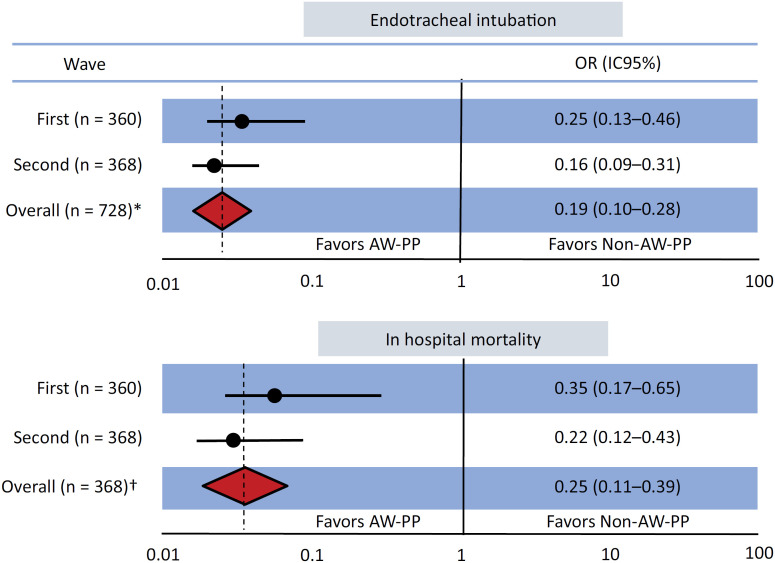
Risk of intubation and in-hospital mortality between groups in the awake prone position and non-prone position during the 1st and 2nd waves in Argentina.

We collected data on the patients’ demographics, comorbidities, severity scores upon ICU admission (Acute Physiology and Chronic Health Evaluation [APACHE II] and Sequential Organ Failure Assessment [SOFA]), chronology of the disease, vital signs, laboratory parameters, PaO_2_/FiO_2_, respiratory rate oxygenation index (ROX index),^(16)^ and chest computed tomography score (CT score).^(17)^ To estimate the level of health care system stress, we assessed the incidence of COVID-19 infection and the ratio of ICU admission/hospital admission rates in the geographic region of each participating center at the time of ICU admission for each subject (Figures 2S and 3S - Supplementary Material). To evaluate the effects of vaccination, we assessed both the population-level vaccination coverage and the vaccination status of each subject at inclusion^(7)^ (Figure 4S - Supplementary Material). Concerning the body position, the following data were recorded: the predominant position adopted by the patient, defined as the position in which the patient spent most hours/day, i.e., prone, lateral, or supine positions; the average number of hours/day in that position; and the number of days of exposure to said position for a period of ≥ 6 hours/day (AW-PP). Awake-prone positioning was defined as remaining in this position for at least 6 hours per day. Patients who did not achieve prone positioning for at least 6 hours were defined as controls.^(13,18)^

### Outcomes

The primary outcome was receiving ETI. The decision to intubate was based on the criteria of the attending health care team. However, intubation was recommended whether specific criteria were met or not: deterioration of neurologic status, hemodynamic instability, or the presence of two or more of the following criteria: a decrease in oxygen saturation with SpO_2_ < 90% for more than 5 minutes (not explained by technical failure), a lack of improvement in the signs of respiratory muscle fatigue, inability to control airway secretions, and respiratory acidosis with pH < 7.30.^(13,15)^ There was no specific position or time limit required before intubation. The secondary outcome was hospital mortality. These outcomes were assessed for the first- and second-wave populations. Additionally, we described other clinical outcomes: ICU mortality, days of ventilatory support, hospital length of stay, and time to ETI.

### Statistical analysis

To evaluate the effectiveness of the AW-PP strategy in each wave, we decided to employ the same statistical methods that had been previously used (Supplementary Material).^(13)^ We used descriptive statistics to describe patients’ baseline characteristics. Standardized mean differences were used to assess the balance between the baseline characteristics of patients who received prone positioning and those of controls (≤ 10%, indicating good balance).^(18)^ Inverse probability of treatment weighting (IPTW) was used to control for potential confounding by indication.^(19,20)^ Direct acyclic graphs (DAGs) were used to identify and select variables potentially associated with both AW-PP and study outcomes^(21,22)^ (Figure 5S and Table 1S - Supplementary Material). First, we created a propensity score by fitting a multivariable logistic regression model with AW-PP as the binary outcome. The following confounders were included (analysis for both waves): age, sex, body mass index (BMI), comorbidities, smoking status, SOFA score, days from symptom onset to hospital admission, previous days of oxygen therapy, previous antibiotic and corticosteroid therapy, use of light sedation, PaO_2_/FiO_2_ and respiratory rate (at inclusion). For the second wave, individual vaccination status was added. Finally, the analysis was performed by fitting logistic regression models with AW-PP as the main exposure and ETI and in-hospital mortality as the dependent variables for the primary and secondary outcomes, respectively. Then, in a logistic regression model for each wave, the proxy variables of expertise acquisition throughout the pandemic (epidemiological week) and health system stress (analysis for both waves) as well as the population vaccination rate (analysis for wave 2) were included to adjust for their possible effect on outcomes ("doubly robust" approach) via the population weighted by the above procedure (IPTW). Measures of association are expressed as odds ratios (ORs) with 95% confidence intervals (95%CIs).^(23)^

In a previous study, we reported an EIT frequency of 23% in AW-PP and 53% in non-prone positioning (Non-PP).^(13)^ Including the total number of patients in each wave, the power to find the same difference with an alpha of 0.05 two-tailed is 100%.

A forest plot was used to display effect estimates and confidence intervals for both individual studies and meta-analyses by common effect inverse variance.^(24)^ To assess magnitude and accuracy, the ORs were evaluated with their 95%CIs.

Given that some of the patients in the Non-AW-PP Group were exposed to the intervention (< 6 hours/day), we performed a restricted analysis comparing AW-PP > 6 hours *versus* patients with "zero" hours in the prone position. Additionally, the prone position has been shown to reduce adverse events in ventilated patients when the PaO_2_/FiO_2_ ratio is < 150mmHg. We performed restricted analyses according to the level of hypoxemia severity (PaO_2_/FiO_2_ < 150mmHg).

Given the likelihood of an unmeasured confounder, we estimated the e-value as a way to determine how strong the association between such a confounder with both exposure and outcome should be to fully explain the estimated effect.^(25)^ Every test was two-sided, and a p value < 0.05 was considered statistically significant. All analyses were performed with STATA version 15.1. For more details about the statistical analysis, see the Supplementary Material.

## RESULTS

During the study period, 1,263 patients with COVID-19-related ARF were admitted to the participating ICUs, 728 of whom met the inclusion criteria (Figure 1). Three hundred and sixty patients were included during the first wave and 368 during the second wave, of whom 195 (54%) and 227 (62%) remained on prone position for a median (p 25 - 75) of 12 (10 - 16) and 14 (8 - 17) hours/day, respectively, and were analyzed in the intervention group (AW-PP Group) (Figure 1). One hundred and sixty-five (46%) and 141 (38%) patients were treated with HFNO but did not complete 6 hours in the AW-PP during the 1st and 2nd waves, respectively, and therefore served as the control group (Non-PP Group). The baseline characteristics of the population are described in table 1 and table 2S (Supplementary Material). The balance between groups in each wave after IPTW is described in table 2. Even though there were differences between the intervention and control groups in the 1st and 2nd waves, after weighting by IPTW, the values of all the variables were balanced, with a standardized mean difference of less than 0.1 (Table 2, and Figure 6SA and B - Supplementary Material).

ARF - acute respiratory failure; ICU - intensive care unit; MV - mechanical ventilation; HFNO - high-flow nasal cannula; PaO_2_ - partial pressure of oxygen; FiO_2_ - fraction of inspired oxygen; AW-PP - awake prone position.

**Table 1:** Baseline characteristics of the study population

Characteristic		1^st^ wave (n = 360)	2^nd^ wave (n = 368)	Overall (n = 728)	p value*
Demographics					
	Age (years)	61 (49 - 71)	56 (46 - 65)	57 (47 - 68)	< 0.001
	Female sex	99 (27)	111 (30)	210 (29)	0.43
	Body mass index	30 (27 - 34)	30 (28 - 35)	30.1 (27 - 34)	0.066
Comorbidities					
	Respiratory	53 (15)	60 (16)	113 (16)	0.56
	Smoking	106 (29)	65 (18)	171 (23)	< 0.001
	Cardiovascular	102 (28)	137 (37)	239 (33)	0.01
	Renal	19 (5)	18 (5)	37 (5)	0.80
	Hepatic	5 (1)	8 (2)	13 (2)	0.43
	Neurologic	13 (4)	19 (5)	32 (4)	0.04
	Diabetes	92 (26)	71 (19)	163 (22)	0.012
	Hypertension	163 (45)	133 (36)	296 (41)	0.012
	Immunosuppression†	29 (8)	17 (5)	46 (6)	0.040
Respiratory-hemodynamics and scores at admission					
	APACHE	10 (7 - 13)	13 (8 - 17)	11 (8 - 15)	< 0.001
	SOFA	3 (2 - 4)	3 (2 - 4)	3 (2 - 4)	0.78
	CT‡	13 (9 - 17)	15 (10 - 19)	14 (10 - 18)	< 0.001
	SpO_2_/FiO_2_	139 (121 - 237)	125 (119 - 139)	133 (120 - 215)	< 0.001
	PaO_2_/FiO_2_	129 (104 - 178)	99 (77 - 133)	123 (86 - 160)	< 0.001
	ROX index	6.3 (4.6 - 9.5)	5 (4.2 - 6.4)	5.6 (3.2 - 7.8)	< 0.001
Chronology of disease					
	Days from symptom onset to hospital admission	8 (5 - 10)	10 (7 - 11)	9 (7 - 11)	< 0.001
	Previous days of oxygen therapy	1 (0 - 3)	1 (0 - 3)	1 (0 - 3)	0.95
Treatments					
	Systemic corticosteroids	356 (99)	362 (98)	718 (99)	0.17
	Light sedation/opioids	170 (47)	261 (71)	431 (59)	< 0.001
	Dexmedethomidine	24 (7)	21 (6)	45 (6)	
	Opioids	44 (12)	58 (16)	102 (14)	
	Dexmedetomidine plus opioids	102 (28)	182 (49)	284 (39)	
	Antibiotics al ICU admission	298 (83)	119 (32)	417 (57)	< 0.001
	Vaccination	-	74 (10)	74 (10)	< 0.001

APACHE - Acute Physiology and Chronic Health Evaluation; SOFA - Sequential Organ Failure Assessment; CT - computed tomography; SpO_2_ - peripheral oxygen saturation; FiO_2_ - fraction of inspired oxygen; PaO_2_ - partial pressure of oxygen; ROX - respiratory rate oxygenation.

* p value, comparison between the 1st and 2nd waves;

† includes oncohematologic or solid neoplasm, drug-induced immunosuppression or other types of immunosuppression;

‡ 142 (19%) patients did not have computed tomography scans at the time of intensive care unit admission. The values were calculated as the mean of each group population. The results are expressed as the median (p 25-75) or n (%).

**Table 2:** Baseline characteristics of the study population after inverse probability of treatment weighting in each wave*

Characteristic		1^st^ wave (n = 360)			2^nd^ wave (n = 368)		
		AW-PP (n = 195)	Non-AW-PP (n = 165)	Standardized difference	AW-PP (n = 227)	Non-AW-PP (n = 141)	Standardized difference
Demographics							
	Age (years)	59.36	58.91	0.03	54.20	53.57	0.05
	Female	0.24	0.27	-0.07	0.27	0.22	-0.10
	Body mass index	31.21	30.95	0.04	32	32.08	-0.01
Comorbidities							
	Respiratory	0.16	0.14	0.06	0.15	0.16	-0.03
	Smoking	0.30	0.29	0.01	0.13	0.13	-0.01
	Cardiovascular	0.26	0.27	-0.01	0.37	0.37	-0.01
	Renal	0.07	0.08	-0.05	0.04	0.04	-0.00
	Neurologic	0.02	0.03	-0.03	0.05	0.05	0.01
	Diabetes	0.30	0.31	-0.02	0.19	0.22	-0.08
	Hypertension	0.45	0.44	0.01	0.33	0.31	0.05
	Immunosuppression†	0.09	0.07	0.08	0.02	0.02	0.00
Respiratory and severity scores at admission							
	SOFA	3.12	3.14	-0.01	2.98	2.93	0.03
	PaO_2_/FiO_2_‡	148.96	144.20	0.08	119.32	118.04	0.03
	Respiratory rate	26.28	26.07	0.04	26.95	27.09	-0.02
Chronology of disease							
	Days from symptom onset to hospital admission	8.03	8.45	-0.10	9.84	10.07	-0.07
	Previous days of oxygen therapy	1.76	1.77	-0.01	1.72	1.74	-0.01
Treatments							
Systemic corticosteroids		0.99	0.99	-0.00	0.98	0.98	0.03
Light sedation		0.47	0.47	-0.00	0.72	0.69	0.07
Antibiotics		0.84	0.85	-0.04	0.29	0.29	-0.01
Vaccination§		-	-	-	0.19	0.14	0.12

AW-PP - awake-prone positioning; SOFA - Sequential Organ Failure Assessment; PaO_2_ - partial pressure of oxygen; FiO_2_ - fraction of inspired oxygen.

* Variables included in the logistic regression model with the awake prone position as the main exposure. The values are expressed as standardized means. A standardized difference > 0.1 represents an imbalance between the groups;

† includes oncohaematologic or solid neoplasm, drug-induced immunosuppression or other types of immunosuppression;

‡ For the inverse probability of treatment weighting model, missing data were imputed in 11% (n = 84) of the records with the mean (122) of the study population;

§ the vaccination programme started during the second wave.

The number of incident cases, the distribution of variants and vaccination coverage during the study period are described in the Supplementary Material. The variables related to oxygen therapy, exposure to prone positioning, and clinical outcomes are listed in table 3.

**Table 3:** Interventions and main outcomes of the study population according to the COVID-19 wave

			1^st^ wave (n = 360)		2^nd^ wave (n = 368)	
			AW-PP (n = 195)	Non-AW-PP (n = 165)	AW-PP (n = 227)	Non-AW-PP (n = 141)
Oxygen therapy and prone positioning						
	Exposure to AW-PP and HFNO					
		Time in AW- PP (hour/day)	12 (10 - 16)	0 (0 - 2)	14 (9 - 18)	4 (4 - 5)
		Time in AW-PP (days)	5 (3 - 8)	0 (0 - 2)	5 (3 - 7)	3 (2 - 5)
		Time on HFNO (days)	5 (3 - 7)	3 (1 - 7)	5 (3 - 7)	3 (1 - 5)
	Basal setting of HFNO					
		FIow (L/minute)	60 (60 - 65)	60 (50 - 60)	60 (50 - 60)	60 (50 - 60)
		FiO_2_	0.6 (0.5 - 0.7)	0.6 (0.5 - 0.75)	0.7 (0.6 - 0.8)	0.7 (0.6 - 0.9)
Main outcomes						
	Endotracheal intubation, overall		41 (21)	86 (52)	60 (26)	80 (57)
		Progression of respiratory failure	39 (95)	83 (96)	58 (97)	79 (99)
		Hemodynamic failure	1 (2)	2 (2)	1 (2)	1 (1)
		Neurologic failure	1 (2)	1 (1)	1 (2)	0
		Cardiac arrest	-	1 (1)	-	-
	Time from start HFNO to intubation (days)		2 (1 - 4)	1 (1 - 3)	4 (3 - 6)	2 (1 - 3)
	Days free of respiratory support*		22 (17 - 24)	19 (10 - 23)	21 (17 - 24)	19 (11 - 23)
	VFDs		28 (28 - 28)	21 (0 - 28)	28 (23 - 28)	20 (0 - 28)
	Time on invasive mechanical ventilation		16 (10 - 25)	13 (8 - 24)	15 (8 - 30)	12 (7 - 27)
	ICU LOS		9 (6 - 14)	12 (7 - 21)	8 (5 - 14)	11 (6 - 26)
	Hospital LOS		15 (11 - 24)	20 (15 - 32)	15 (11 - 22)	18 (11 - 34)
	In-hospital mortality		18 (9)	54 (33)	32 (14)	37 (26)
	Mortality at ICU discharge		19 (10)	54 (33)	32 (14)	37 (26)

AW-PP - awake prone position; HFNO - high-flow nasal oxygen; FiO_2_ - fraction of inspired oxygen; VFDs - ventilator-free days, on Day 28; ICU - intensive care unit; LOS - length of stay.

* Including high-flow nasal oxygen, noninvasive and invasive ventilation. The results are expressed as the median (p 25-75) or n (%).

During the first wave, 41 (21%) patients in the AW-PP Group *versus* 86 (52%) in the Non-PP Group were intubated, whereas during the second wave, 60 (26%) *versus* 80 (57%) patients were intubated. Overall, 101 (24%) patients in the AW-PP Group *versus* 166 (53%) in the Non-PP Group were intubated. In the weighted and adjusted population, the OR for ETI was 0.19 (95%CI 0.10 - 0.28) during the 1st wave and 0.16 (95%CI 0.09 - 0.31) during the 2nd wave (Figure 2). In the overall weighted and adjusted population, the OR for ETI was 0.19 (95%CI 0.10 - 0.28). No differences were observed between the two waves in terms of effect size (p = 0.40) (Figure 2). The e-value for the primary analysis of the effects of AW-PP on intubation was 3.41 for the first wave and 4.44 for the second wave (Figure 7S - Supplementary Material).

OR - odds ratio; 95%CI - 95%confidence interval; AW-PP - awake prone positioning. The Nonawake Prone Positioning Group was used as a reference. The population was weighted and adjusted by center, pandemic time (epidemiological week), health care system stress (both waves) and the population vaccination rate (2nd wave). * I^2^ = 0.0%, p = 0.401; † I^2^ = 0.0%, p = 0.440.

During the first wave, 19 (10%) patients in the AW-PP Group *versus* 54 (33%) in the Non-PP Group died in the hospital, whereas during the second wave, 32 (14%) *versus* 37 (26%) patients died, respectively (Table 3). Overall, 51 (12%) patients in the AW-PP Group *versus* 91 (30%) in the Non-PP Group died. In the weighted and adjusted population, the OR for hospital mortality was 0.35 (95%CI 0.17 - 0.65) during the 1st wave and 0.22 (95%CI 0.12 - 0.43) during the 2nd wave (Figure 2). In the overall weighted and adjusted population, the OR for hospital mortality was 0.25 (95%CI 0.11 - 0.39) and no differences were observed between the two waves (p = 0.44) (Figure 2).

The reasons for ETI in the 267 patients were the progression of respiratory failure [n = 259 (97%)] and hemodynamic failure [n = 5 (2%)] (Table 3). The effects of exposure to AW-PP on intubation remained when they were evaluated according to the severity of respiratory failure defined by a PaO_2_/FiO_2_ ≤ 150 during the 1st and 2nd waves [OR = 0.25 (0.11 - 0.56), and 0.14 (0.07 - 0.26) for the 1^st^ and 2^nd^ waves, respectively] (Table 3S - Supplementary Material). The results of the analysis restricted to patients with AW-PP > 6 hours *versus* patients with "zero" hours in the prone position (n = 313) on ETI showed a consistently reduced risk of intubation in both waves [OR = 0.32 (0.17 - 0.62) and 0.19 (0.10 - 0.33) for the 1st and 2nd waves, respectively] (Table 3S - Supplementary Material).

The main clinical outcomes are displayed in table 3. The ICU and hospital lengths of stay as well as the number of ICU deaths were lower, and the number of days free of respiratory support (including HFNO) was greater in the AW-PP Group, overall and during the 1st and 2nd waves.

## DISCUSSION

In this multicenter, prospective observational study of patients with COVID-19-related ARF receiving initial treatment with HFNO, AW-PP for at least 6 hours a day was associated with a lower risk of ETI and hospital mortality, even after adjustment for potential confounders. These effects were observed in the entire cohort of patients and during each of the periods evaluated, without differences between the first and second waves in Argentina.

The COVID-19 pandemic has led to a unique health crisis in recent history. During this period, epidemiological variables, the characteristics of infected patients, the course of the disease, interventions, the prognosis and the clinical outcomes of patients constantly changed.^(26)^ This unique and changing situation makes it necessary to evaluate the effectiveness of treatments in different contexts.^(27)^ The AW-PP has been shown to be effective in reducing ETI in patients with COVID-19-related ARF requiring HFNO when performed in ICUs.^(4,28)^ This evidence was generated mainly during the first stage of the pandemic.^(4,29)^

In our study, HFNO and AW-PP were applied concomitantly and systematically in a cohort of consecutive patients. Since the study groups displayed differences in baseline variables, a causal approximation was carried out by weighting these variables via the propensity score method (IPTW). The results revealed a balanced distribution of variable values between groups (in both waves), thereby allowing the assumption of population interchangeability.^(18)^ Variables included in the model were selected by the DAGs and were individual- or patient-specific. However, the effectiveness of interventions over time may also be influenced by not only individuals but also contextual variables.^(30,31)^ Therefore, after weighting by IPTW, we adjusted for these contextual variables. High incidence rates of COVID-19 and stress in health care systems have been associated with increased mortality.^(6,26,32)^ To account for these issues, we adjusted for the incidence rate of COVID-19 and the ICU admission/hospital admission ratio in each participating center region at the time of patient admission. SARS-CoV-2 has changed significantly in terms of its genetic sequence, giving rise to different variants which may potentially play a role in clinical outcomes.^(31)^ In Argentina, the first wave was dominated by the wild variant of the virus, whereas in the second wave, the gamma variant prevailed.^(33)^ On the other hand, health care teams may have acted differently as they gained experience over the course of the pandemic.^(6,26)^ To address these issues, we adjusted for the time of the pandemic in which patients were included. The general effects of vaccination depend on both the direct effects on vaccinated individuals and the indirect effects on unvaccinated individuals.^(34)^ To account for these factors, we included individual vaccination status and then adjusted for the rate of the population vaccinated at the time of ICU admission over the weighted population. The effect of exposure (AW-PP) on clinical outcomes assessed by causal inference (IPTW) with further adjustment for contextual variables showed consistent results in each of the waves. This suggests that the effects of the intervention are independent of the context in which it is applied. Importantly, the efficacy of non-pharmacological treatments may undergo modifications over time, influenced by changes in contextual factors that affect clinical outcomes. In a cohort study aimed at investigating the outcomes of 1,345 patients who received extracorporeal membrane oxygenation for ARDS due to COVID-19 over 4 semesters (2020 - 2021), Schmidt et al. reported that 90-day mortality was 42%. Mortality was 10% higher in the second semester of 2020 and was independently associated with the delta variant than with the other variants.^(35)^

Although some clinical trials have shown that reducing the risk of ETI in patients with COVID-19-related ARF, no study has shown a reduction in the risk of mortality.^(3,4)^ However, these results are limited by the short exposure time achieved in all studies. The time of exposure to AW-PP appears to be a key issue for its effectiveness.^(13,28,36)^ The rate of adherence in our study was high, more than double the exposure times reported in randomized clinical trials.^(4)^ These rates could be explained by several factors, including the interventions being performed in ICUs (where observation, monitoring, and patient instructions can be carried out more effectively) and the use of light sedation opioids or both in more than half of the patients. These times could explain the effectiveness in terms of the reduced risk of ETI and in-hospital mortality attained in the two waves. Remarkably, although the characteristics of the first- and second-wave populations were different in our study, the effectiveness of the intervention evaluated was not significantly different in terms of effect size.

Our study has several strengths: a representative sample of patients with COVID-19-related ARF from different ICUs with consecutive inclusion criteria and similar initial treatment (HFNO), thus minimizing selection bias, and exhaustive treatment of confounders via causal inference. The main limitations of this study are those inherent to establishing causality via an observational design, i.e., the possibility of not considering unmeasured confounders. Several strategies were employed to minimize the potential biases inherent to the design: the prospective nature of the cohort allowed us to consider most of the known confounders; the use of IPTW in the adjustment permitted us to reduce multidimensionality and balance the factors that could influence the hypothesis;^(23)^ additionally, the estimation of the e-value was robust to potential unmeasured confounders.^(25)^ However, it is necessary to emphasize that propensity methods address only observed bias (not unmeasured confounders).^(37)^ Additionally, even when we account for confounders, we can never truly ensure that we sufficiently capture all the needed information given that variables may not possess adequate granularity.^(37)^ Finally, another limitation was the potential bias derived from the non-blinded position of the attending team to the intubation order. Although the health care team followed the recommendations preestablished in the protocol, we did not record the specific reason for intubation for each patient. Importantly, the results for all-cause mortality, an outcome less susceptible to bias, were consistent with the primary outcome (ETI), supporting the hypothesis about the potential benefit of the intervention. All the patients included were treated in an ICU setting. Therefore, these results cannot be extrapolated to patients seen in a less complex setting or with less severe disease.

## CONCLUSION

In patients with COVID-19 and acute respiratory failure admitted to the intensive care unit and initially treated with high-flow nasal oxygen, prone positioning was associated with a reduction in the risk of endotracheal intubation and hospital mortality. These effects were independent of the context in which the intervention was applied, and no differences were observed between the different waves.
