# Supplementary material for: Comparison of the effectiveness of awake-prone positioning and high-flow nasal oxygen in patients with COVID-19-related acute respiratory failure between different waves
Source: Crit Care Sci. 2024 Oct 9;36:e20240065en. doi: 10.62675/2965-2774.20240065-en (PMC11484112; doi:10.62675/2965-2774.20240065-en)
Supplement: Translation

# Translation: Comparação da eficácia da posição prona consciente e oxigênio nasal de alto fluxo em pacientes com insuficiência respiratória aguda relacionada à COVID-19 em diferentes ondas

**DOI:** 10.62675/2965-2774.20240065-pt

**Authors:** Mariano Esperatti, Matías Olmos, Marina Busico, Adrian Gallardo, Alejandra Vitali, Jorgelina Quintana, Hiromi Kakisu, Bruno Leonel Ferreyro, Nora Angélica Fuentes, Argentine Collaborative Group on High Flow and Prone Positioning, Javier Osatnik, Santiago Nicolas Saavedra, Agustin Matarrese, Greta Dennise Rebaza Niquin, Elizabeth Gisele Wasinger, Giuliana Mast, Facundo Juan Andrada, Ana Inés Lagazio, Nahuel Esteban Romano, Marisol Mariela Laiz, Jose Garcia Urrutia, Mariela Adriana Mogaadouro, Micaela Ruiz Seifert, Emilce Mastroberti, Claudia Navarro Moreno, Anabel Miranda Tirado, María Constanza Viñas, Juan Manuel Pintos, Maria Eugenia Gonzalez, Maite Mateos, Verónica Barbaresi, Ana Elizabeth Grimbeek, Leonel Stein, Ariel Juan Latronico, Silvia Laura Menéndez, Alejandra Dominga Basualdo, Romina Castrillo

**Affiliations:** 1 Mar del Plata Argentina Departamento de Terapia Intensiva, Hospital Privado de Comunidad, Escuela Superior de Medicina, Universidad Nacional de Mar del Plata - Mar del Plata, Argentina.; 2 Buenos Aires Argentina Unidade de Terapia Intensiva, Clínica Olivos, Swiss Medical Group - Buenos Aires, Argentina.; 3 Buenos Aires Argentina Unidade de Terapia Intensiva, Sanatorio Clínica Modelo de Morón - Buenos Aires, Argentina.; 4 Buenos Aires Argentina Unidade de Terapia Intensiva, Sanatorio de la Trinidad Palermo - Buenos Aires, Argentina.; 5 Toronto Canada Divisão Interdepartamental de Medicina de Cuidados Críticos, University of Toronto - Toronto, Canada.; 6 Intensive Care Unit Hospital Alemão Buenos Aires Argentina Intensive Care Unit, Hospital Alemão - Buenos Aires, Argentina.; 7 Intensive Care Unit Hospital Universitario Austral Buenos Aires Argentina Intensive Care Unit, Hospital Universitario Austral - Buenos Aires, Argentina.; 8 Intensive Care Unit Clínica Olivos Buenos Aires Argentina Intensive Care Unit, Clínica Olivos - Buenos Aires, Argentina.; 9 Intensive Care Unit Sanatorio de la Trinidad Palermo Buenos Aires Argentina Intensive Care Unit, Sanatorio de la Trinidad Palermo - Buenos Aires, Argentina.; 10 Intensive Care Department Hospital Privado de Comunidad Mar del Plata Argentina Intensive Care Department, Hospital Privado de Comunidad - Mar del Plata, Argentina.

**Keywords:** Insuficiência respiratória, COVID-19, Infecções por coronavírus, SARS-CoV-2, Intubação endotraqueal, Mortalidade hospitalar, Prona, Oxigênio

## Abstract

**Objetivo:**

Comparar a eficácia da posição prona consciente nos desfechos clínicos relevantes em pacientes com insuficiência respiratória aguda relacionada à COVID-19 que necessitaram de oxigênio nasal de alto fluxo em diferentes ondas da doença na Argentina.

**Métodos:**

Trata-se de estudo de coorte prospectivo e multicêntrico, que incluiu pacientes adultos com insuficiência respiratória aguda relacionada à COVID-19 que necessitaram de oxigênio nasal de alto fluxo. A principal posição de exposição foi a prona consciente (≥ 6 horas/dia) em comparação com a prona não consciente. O desfecho primário foi a intubação endotraqueal, e o secundário foi a mortalidade hospitalar. Utilizou-se o escore de propensão de ponderação pela probabilidade inversa para ajustar a probabilidade condicional de atribuição de tratamento. Em seguida, ajustamos as variáveis contextuais, que variaram ao longo do tempo e comparamos a eficácia entre a primeira e a segunda onda.

**Resultados:**

Ao todo, 728 pacientes foram incluídos: 360 durante a primeira onda e 368 durante a segunda onda, dos quais 195 (54%) e 227 (62%) permaneceram em prona consciente numa mediana (intervalo interquatil 25 - 75%) de 12 (10 - 16) e 14 (8 - 17) horas/dia, respectivamente (Grupo Prona Consciente). As razões de chance (intervalo de confiança de 95%) de intubação endotraqueal no grupo prona em vigília foram de 0,25 (0,13 - 0,46) e 0,19 (0,09 - 0,31) na primeira e segunda ondas, respectivamente (p = 0,41 comparativamente entre as ondas). As razões de chance de mortalidade intra-hospitalar na posição prona consciente foram de 0,35 (0,17 - 0,65) e 0,22 (0,12 - 0,43), respectivamente (p = 0,44 comparativamente entre as ondas).

**Conclusão:**

A posição prona consciente foi associada à redução no risco de intubação endotraqueal e mortalidade hospitalar. Esses efeitos foram independentes do contexto em que a intervenção foi aplicada e não foram observadas diferenças entre as diferentes ondas.

## INTRODUÇÃO

Um subgrupo de pacientes com a doença pelo coronavírus 2019 (COVID-19) desenvolve insuficiência respiratória aguda e síndrome do desconforto respiratório agudo (SDRA) e requer ventilação mecânica invasiva.^(1)^ Essa condição está associada a taxas de mortalidade superiores a 40% em países desenvolvidos e em desenvolvimento.^(2)^ Foi demonstrado que a posição prona consciente (PP-C) reduz o risco de intubação endotraqueal (IET) em pacientes com insuficiência respiratória aguda (IRA) relacionada à COVID-19 que recebem suporte respiratório avançado não invasivo no ambiente da unidade de terapia intensiva (UTI).^(3,4)^ As evidências que demonstram a eficácia da PP-C provêm de estudos realizados principalmente durante o estágio inicial da pandemia.^(4)^ Muitos aspectos mudaram durante o curso da pandemia. A incidência e a taxa de internações variaram, desafiando os sistemas de saúde a lidar com diferentes níveis de estresse.^(5,6)^ A cobertura vacinal também mudou ao longo do tempo em diferentes sistemas de saúde.^(7,8)^ Da mesma forma, o coronavírus da síndrome respiratória aguda grave 2 (SARS-CoV-2) apresenta múltiplas variações em sua sequência genética, levando ao desenvolvimento de novas ondas da COVID-19.^(9-11)^ Embora essas variáveis possam influenciar o desfecho clínico de pacientes com COVID-19, os tratamentos que se mostraram eficazes não foram avaliados quanto à eficácia durante toda a pandemia, nem seus efeitos foram comparados em diferentes momentos durante seu curso.

No presente estudo, nosso objetivo foi comparar a eficácia da PP-C nos desfechos clínicos relevantes em pacientes com IRA relacionada à COVID-19 que necessitaram de oxigênio nasal de alto fluxo em diferentes ondas da doença na Argentina. Nossa hipótese foi considerar que o efeito do tratamento poderia variar ao longo do tempo devido às múltiplas variáveis que sofreram alterações ao longo de seu curso.

## MÉTODOS

Realizamos um estudo de coorte prospectivo e multicêntrico em seis UTIs de seis centros na Argentina. O estudo foi registrado no ClinicalTrials.gov (NCT05178212) e relatado conforme as diretrizes STROBE (Material Suplementar).^(12)^ Os comitês de revisão interna dos seis centros aprovaram o estudo, e dispensou-se o Termo de Consentimento Livre e Esclarecido (Material Suplementar). As intervenções realizadas faziam parte da prática habitual de cada centro, e os pesquisadores garantiram a confidencialidade das informações de seus pacientes. Já foi relatada uma descrição detalhada dos métodos, dos procedimentos relacionados à intervenção avaliada e da análise estatística (Material Suplementar).^(13)^

### Curso da pandemia e contexto do estudo (Material Suplementar)

O período do estudo abrangeu de março de 2020 a setembro de 2021. A primeira onda foi considerada como tendo ocorrido de 27 de fevereiro de 2020 a 16 de fevereiro de 2021, sendo que os pacientes incluídos após esse período corresponderam à segunda onda.^(14)^ A figura 1S (Material Suplementar) apresenta as variantes predominantes identificadas durante esse período.

### População do estudo

Incluímos consecutivamente pacientes a partir de 18 anos admitidos na UTI com diagnóstico confirmado da COVID-19 e recebendo oxigênio nasal de alto fluxo (ONAF) por pelo menos 4 horas. Os pacientes receberam ONAF na presença de quaisquer dos seguintes critérios: saturação periférica de oxigênio (SpO_2_) < 92% com oxigênio > 4L/minuto; aumento do trabalho respiratório com o uso de músculos respiratórios acessórios e frequência respiratória > 30/minuto; e relação entre pressão parcial de oxigênio/fração inspirada de oxigênio (PaO_2_/FiO_2_) < 200mmHg. Foram excluídos os pacientes com nível de consciência diminuído, presença de choque, necessidade imediata de intubação,^(15)^ uso de ventilação com pressão positiva antes do ONAF ou ordem de não intubar.

### Procedimento do estudo (Material Suplementar)

Imediatamente após a admissão na UTI, avaliaram-se os critérios de aptidão e iniciou-se o ONAF com fluxo entre 50 e 70L/minuto, com a FiO_2_ mínima necessária para se obter SpO_2_ > 92%. Incluíram-se os pacientes que toleraram o ONAF nas 4 horas seguintes. A equipe de assistência médica incentivou e auxiliou todos os participantes a passarem da posição supina para a prona pelo maior tempo possível, fazendo pausas para higiene pessoal e alimentação. Quando os pacientes não toleravam a posição prona, recebiam auxílio para permanecer na posição lateral, alternando entre os decúbitos direito e esquerdo, pelo tempo que pudessem tolerar. Quando os pacientes não toleravam nenhuma das posições mencionadas, permitia-se a posição supina. Essas intervenções foram mantidas durante o período do estudo até que se atendesse a um dos seguintes critérios: manutenção da SpO_2_ > 92% com FiO_2_ ≤ 40%, fluxo ≤ 40L/minuto por um período > 12 horas na posição supina ou IET. Uma vez obtida a estabilidade clínica e gasosa com o ONAF, o fluxo e o oxigênio eram progressivamente desmamados, com base num protocolo (Material Suplementar).^(13)^ Drogas analgésicas (opioides, paracetamol) ou sedação leve (dexmedetomidina) eram permitidas e indicadas conforme os critérios da equipe médica (Material Suplementar).

### Variáveis e avaliações

Coletamos dados demográficos dos pacientes, das comorbidades, escores de gravidade na admissão na UTI (*Acute Physiology and Chronic Health Evaluation* [APACHE II] e *Sequential Organ Failure Assessment* [SOFA]), cronologia da doença, sinais vitais, parâmetros laboratoriais, PaO_2_/FiO_2_, índice de oxigenação da frequência respiratória (índice ROX)^(16)^ e escore da tomografia computadorizada de tórax (escore TC).^(17)^ Para estimar o nível de estresse do sistema de assistência médica, avaliamos a incidência de infecção por COVID-19 e a proporção das taxas de admissão na UTI/admissão hospitalar na região geográfica de cada centro participante no momento da admissão na UTI de cada indivíduo (Figuras 2S e 3S - Material Suplementar). Para avaliar os efeitos da vacinação, avaliamos a cobertura de vacinação em nível populacional e o histórico de vacinação de cada indivíduo no momento da inclusão^(7)^ (Figura 4S - Material Suplementar). Com relação à posição do corpo, registramos os seguintes dados: a posição predominante adotada pelo paciente, definida como a posição em que o paciente passou a maior parte das horas/dia, ou seja, as posições prona, lateral ou supina; o número médio de horas/dia nessa posição; e o número de dias de exposição a essa posição por um período ≥ 6 horas/dia (PP-C). Definiu-se PP-C como a permanência nessa posição por pelo menos 6 horas por dia. Os pacientes que não permaneceram na posição prona por pelo menos 6 horas foram definidos como controles.^(13,18)^

### Desfechos

O desfecho primário foi a IET. A decisão de intubação ocorreu com base nos critérios da equipe médica responsável. No entanto, a intubação foi recomendada independentemente do cumprimento ou não de critérios específicos: deterioração do estado neurológico, instabilidade hemodinâmica ou presença de dois ou mais dos seguintes critérios: diminuição da saturação de oxigênio, com SpO_2_ < 90% por mais de 5 minutos (não explicada por falha técnica); ausência de melhora nos sinais de fadiga muscular respiratória; incapacidade de controlar as secreções das vias aéreas; e acidose respiratória, com pH < 7,30.^(13,15)^ Não foi exigida uma posição específica ou limite de tempo antes da intubação. O desfecho secundário foi a mortalidade hospitalar. Esses resultados foram avaliados nas populações da primeira e da segunda onda. Além disso, descrevemos outros desfechos clínicos: mortalidade na UTI, dias de suporte ventilatório, duração da internação hospitalar e duração da IET.

### Análise estatística

Na avaliação da eficácia da estratégia PP-C em cada onda, decidimos empregar os mesmos métodos estatísticos que tinham sido usados anteriormente (Material Suplementar).^(13)^ Usamos estatísticas descritivas para descrever as características basais dos pacientes. Usamos diferenças médias padronizadas para avaliar o equilíbrio entre as características basais dos pacientes que receberam PP-C e as dos controles (≤ 10%, indicando bom equilíbrio).^(18)^ Empregou-se ponderação pela probabilidade inversa de tratamento (IPTW - *inverse-probability-of-treatment weighting*) para controlar a possível confusão por indicação.^(19,20)^ Utilizamos gráficos acíclicos diretos (DAGs - *directed acyclic graph*) para identificar e selecionar variáveis potencialmente associadas tanto à PP-C quanto aos desfechos do estudo.^(21,22)^ (Figura 5S e Tabela 1S - Material Suplementar). Primeiramente, criamos um escore de propensão ajustando um modelo de regressão logística multivariável com a PP-C como desfecho binário. Foram incluídos os seguintes fatores de confusão (análise em ambas as ondas): idade, sexo, índice de massa corporal (IMC), comorbidades, tabagismo, pontuação SOFA, dias do início dos sintomas até a admissão hospitalar, dias anteriores de oxigenoterapia, antibioticoterapia e corticoterapia anteriores, uso de sedação leve, PaO_2_/FiO_2_ e frequência respiratória (na inclusão). Na segunda onda, foi adicionado o histórico de vacinação do indivíduo. Por fim, a análise foi realizada por meio do ajuste de modelos de regressão logística com PP-C como exposição principal e IET e mortalidade hospitalar como variáveis dependentes dos desfechos primário e secundário, respectivamente. Em seguida, em um modelo de regressão logística de cada onda, incluíram-se as variáveis *proxy* de aquisição de conhecimento especializado durante a pandemia (semana epidemiológica) e estresse do sistema de saúde (análise de ambas as ondas), bem como a taxa de vacinação da população (análise da segunda onda) para ajustar seu possível efeito sobre os desfechos (abordagem "duplamente robusta") por meio da população ponderada pelo procedimento acima (IPTW). As medidas de associação são expressas como razões de chances (RCs) com intervalos de confiança de 95% (IC95%).^(23)^

Em um estudo anterior, relatamos uma frequência de IET de 23% em PP-C e 53% em posição não prona (Não PP-C).^(13)^ Considerando o número total de pacientes em cada onda, o poder de encontrar a mesma diferença com alfa de 0,05 bicaudal é de 100%.

Utilizou-se gráfico de floresta para exibir estimativas de efeito e intervalos de confiança tanto para estudos individuais quanto para metanálises por variância inversa de efeito comum.^(24)^ Para avaliar a magnitude e a precisão, as RCs foram avaliadas com seus IC95%.

Considerando que alguns dos pacientes do grupo Não PP-C foram expostos à intervenção (< 6 horas/dia), realizamos uma análise restrita comparando PP-C > 6 horas *versus* pacientes com "zero" horas em PP. Além disso, demonstrou-se que a PP reduz os eventos adversos em pacientes ventilados quando PaO_2_/FiO_2_ é < 150mmHg. Realizamos análises restritas de acordo com o nível de gravidade da hipoxemia (PaO_2_/FiO_2_ < 150mmHg).

Dada a probabilidade de um fator de confusão não medido, estimamos o valor e uma forma de determinar o quão forte deve ser a associação entre esse fator de confusão com a exposição e o desfecho, para explicar totalmente o efeito estimado.^(25)^ Todos os testes foram bicaudais, e valor de p < 0,05 foi considerado estatisticamente significativo. Todas as análises foram realizadas com o STATA 15.1. Para mais detalhes da análise estatística, consulte o Material Suplementar.

## RESULTADOS

Durante o período do estudo, foram admitidos 1.263 pacientes com IRA associada à COVID-19 nas UTIs participantes, dos quais 728 atenderam aos critérios de inclusão (Figura 1). Foram incluídos 368 pacientes durante a primeira onda e 368 durante a segunda onda, dos quais 195 (54%) e 227 (62%) permaneceram em posição prona em uma mediana (IQ25 - 75%) de 12 (10 - 16) e 14 (8 - 17) horas/dia, respectivamente, sendo analisados no grupo intervenção (Grupo PP-C) (Figura 1). Receberam ONAF 165 (46%) e 141 (38%) pacientes, mas não completaram 6 horas na PP-C durante a primeira e segunda ondas, respectivamente, e, portanto, fizeram parte do grupo controle (Grupo Não PP-C). As características basais da população estão descritas na tabela 1 e na tabela 2S (Material Suplementar). O equilíbrio entre os grupos em cada onda após o IPTW está descrito na tabela 2. Embora tenha havido diferenças entre os grupos intervenção e controle na primeira e segunda ondas, após a ponderação pelo IPTW, os valores de todas as variáveis foram equilibrados, com diferença média padronizada inferior a 0,1 (Tabela 2 e Figura 6SA e B - Material Suplementar).

**Figura 1:** Inclusão de pacientes.

IRA - insuficiência respiratória aguda; UTI - unidade de terapia intensiva; VM - ventilação mecânica; ONAF - oxigênio nasal de alto fluxo; PaO_2_ - pressão parcial de oxigênio; FiO_2_ - fração inspirada de oxigênio; PP-C - posição prona consciente.

**Tabela 1:** Características basais da população do estudo

Característica		1ª onda (n = 360)	2ª onda (n = 368)	Total (n = 728)	Valor de p*
Dados demográficos					
	Idade (anos)	61 (49 - 71)	56 (46 - 65)	57 (47 - 68)	< 0,001
	Sexo feminino	99 (27)	111 (30)	210 (29)	0,43
	Índice de massa corporal	30 (27 - 34)	30 (28 - 35)	30,1 (27 - 34)	0,066
Comorbidades					
	Respiratórias	53 (15)	60 (16)	113 (16)	0,56
	Tabagismo	106 (29)	65 (18)	171 (23)	< 0,001
	Cardiovasculares	102 (28)	137 (37)	239 (33)	0,01
	Renais	19 (5)	18 (5)	37 (5)	0,80
	Hepáticas	5 (1)	8 (2)	13 (2)	0,43
	Neurológicas	13 (4)	19 (5)	32 (4)	0,04
	Diabetes	92 (26)	71 (19)	163 (22)	0,012
	Hipertensão	163 (45)	133 (36)	296 (41)	0,012
	Imunossupressão†	29 (8)	17 (5)	46 (6)	0,040
Hemodinâmica respiratória e escores na admissão					
	APACHE	10 (7 - 13)	13 (8 - 17)	11 (8 - 15)	< 0,001
	SOFA	3 (2 - 4)	3 (2 - 4)	3 (2 - 4)	0,78
	TC‡	13 (9 - 17)	15 (10 - 19)	14 (10 - 18)	< 0,001
	SpO_2_/FiO_2_	139 (121 - 237)	125 (119 - 139)	133 (120 - 215)	< 0,001
	PaO_2_/FiO_2_	129 (104 - 178)	99 (77 - 133)	123 (86 - 160)	< 0,001
	Índice ROX	6,3 (4,6 - 9,5)	5 (4,2 - 6,4)	5,6 (3,2 - 7,8)	< 0,001
Cronologia da doença					
	Dias do início dos sintomas até a admissão hospitalar	8 (5 - 10)	10 (7 - 11)	9 (7 - 11)	< 0,001
	Dias anteriores de oxigenoterapia	1 (0 - 3)	1 (0 - 3)	1 (0 - 3)	0,95
Tratamentos					
	Corticosteroides sistêmicos	356 (99)	362 (98)	718 (99)	0,17
	Sedação leve/opioides	170 (47)	261 (71)	431 (59)	< 0,001
	Dexmedetomidina	24 (7)	21 (6)	45 (6)	
	Opioides	44 (12)	58 (16)	102 (14)	
	Dexmedetomidina e opioides	102 (28)	182 (49)	284 (39)	
	Antibióticos na admissão na UTI	298 (83)	119 (32)	417 (57)	< 0,001
	Vacinação	-	74 (10)	74 (10)	< 0,001

APACHE - *Acute Physiology and Chronic Health Evaluation*; SOFA - *Sequential Organ Failure Assessment*; TC - tomografia computadorizada; SpO_2_ - saturação periférica de oxigênio; FiO_2_ - fração inspirada de oxigênio; PaO_2_ - pressão parcial de oxigênio; ROX - frequência respiratória de oxigenação.

* Valor de p, comparação entre a primeira e a segunda ondas;

† inclui neoplasia onco-hematológica ou sólida, imunossupressão induzida por medicamentos ou outros tipos de imunossupressão;

‡ 142 (19%) pacientes não tinham exames de tomografia computadorizada na admissão na unidade de terapia intensiva. Os valores foram calculados como a média da população de cada grupo. Resultados expressos como mediana (intervalo interquartil 25% - 75%) ou n (%).

**Tabela 2:** Características basais da população do estudo após a ponderação pela probabilidade inversa de tratamento em cada onda*

Característica		1ª onda (n = 360)			2ª onda (n = 368)		
		PP-C (n = 195)	Não PP-C (n = 165)	Diferença padronizada	PP-C (n = 227)	Não PP-C (n = 141)	Diferença padronizada
Dados demográficos							
	Idade (anos)	59,36	58,91	0,03	54,20	53,57	0,05
	Sexo feminino	0,24	0,27	-0,07	0,27	0,22	-0,10
	Índice de massa corporal	31,21	30,95	0,04	32	32,08	-0,01
Comorbidades							
	Respiratórias	0,16	0,14	0,06	0,15	0,16	-0,03
	Tabagismo	0,30	0,29	0,01	0,13	0,13	-0,01
	Cardiovasculares	0,26	0,27	-0,01	0,37	0,37	-0,01
	Renais	0,07	0,08	-0,05	0,04	0,04	-0,00
	Neurológicas	0,02	0,03	-0,03	0,05	0,05	0,01
	Diabetes	0,30	0,31	-0,02	0,19	0,22	-0,08
	Hipertensão	0,45	0,44	0,01	0,33	0,31	0,05
	Imunossupressão†	0,09	0,07	0,08	0,02	0,02	0,00
Escores respiratórios e de gravidade na admissão							
	SOFA	3,12	3,14	-0,01	2,98	2,93	0,03
	PaO_2_/FiO_2_‡	148,96	144,20	0,08	119,32	118,04	0,03
	Frequência respiratória	26,28	26,07	0,04	26,95	27,09	-0,02
Cronologia da doença							
	Dias do início dos sintomas até a admissão hospitalar	8,03	8,45	-0,10	9,84	10,07	-0,07
	Dias anteriores de oxigenoterapia	1,76	1,77	-0,01	1,72	1,74	-0,01
Tratamentos							
	Corticosteroides sistêmicos	0,99	0,99	-0,00	0,98	0,98	0,03
	Sedação leve	0,47	0,47	-0,00	0,72	0,69	0,07
	Antibióticos	0,84	0,85	-0,04	0,29	0,29	-0,01
	Vacinação§	-	-	-	0,19	0,14	0,12

PP-C - posição prona consciente; SOFA - *Sequential Organ Failure Assessment*; PaO_2_ - pressão parcial de oxigênio; FiO_2_ - fração inspirada de oxigênio.

* Variáveis incluídas no modelo de regressão logística com posição prona em vigília como exposição principal. Os valores são expressos como médias padronizadas. Uma diferença padronizada > 0,1 representa um desequilíbrio entre os grupos;

† inclui neoplasia onco-hematológica ou sólida, imunossupressão induzida por medicamentos ou outros tipos de imunossupressão;

‡ no modelo de ponderação pela probabilidade inversa de tratamento, os dados ausentes foram imputados em 11% (n = 84) dos registros com a média (122) da população do estudo;

§ o programa de vacinação começou durante a segunda onda.

O número de casos incidentes, a distribuição das variantes e a cobertura vacinal durante o período do estudo estão descritos no Material Suplementar. As variáveis relacionadas à ONAF, à posição prona e aos desfechos clínicos estão listadas na tabela 3.

**Tabela 3:** Intervenções e principais desfechos na população do estudo em relação à onda da COVID-19

			1ª onda (n = 360)		2ª onda (n = 368)	
			PP-C (n = 195)	Não PP-C (n = 165)	PP-C (n = 227)	Não PP-C (n = 141)
Oxigenoterapia e posição prona						
	Exposição à PP-C e à ONAF					
		Duração da PP-C (horas/dias)	12 (10 - 16)	0 (0 - 2)	14 (9 - 18)	4 (4 - 5)
		Duração da PP-C (dias)	5 (3 - 8)	0 (0 - 2)	5 (3 - 7)	3 (2 - 5)
		Duração da ONAF (dias)	5 (3 - 7)	3 (1 - 7)	5 (3 - 7)	3 (1 - 5)
	Configuração basal da ONAF					
		Fluxo (L/minuto)	60 (60 - 65)	60 (50 - 60)	60 (50 - 60)	60 (50 - 60)
		FiO_2_	0,6 (0,5 - 0,7)	0,6 (0,5 - 0,75)	0,7 (0,6 - 0,8)	0,7 (0,6 - 0,9)
Principais desfechos						
	Intubação endotraqueal, total		41 (21)	86 (52)	60 (26)	80 (57)
		Progressão da insuficiência respiratória	39 (95)	83 (96)	58 (97)	79 (99)
		Insuficiência hemodinâmica	1 (2)	2 (2)	1 (2)	1 (1)
		Insuficiência neurológica	1 (2)	1 (1)	1 (2)	0
		Parada cardíaca	-	1 (1)	-	-
	Tempo do início da ONAF até a intubação (dias)		2 (1 - 4)	1 (1 - 3)	4 (3 - 6)	2 (1 - 3)
	Dias sem suporte respiratório*		22 (17 - 24)	19 (10 - 23)	21 (17 - 24)	19 (11 - 23)
	DSVs		28 (28 - 28)	21 (0 - 28)	28 (23 - 28)	20 (0 - 28)
	Tempo em ventilação mecânica invasiva		16 (10 - 25)	13 (8 - 24)	15 (8 - 30)	12 (7 - 27)
	Duração da internação na UTI		9 (6 - 14)	12 (7 - 21)	8 (5 - 14)	11 (6 - 26)
	Duração da internação hospitalar		15 (11 - 24)	20 (15 - 32)	15 (11 - 22)	18 (11 - 34)
	Mortalidade intra-hospitalar		18 (9)	54 (33)	32 (14)	37 (26)
	Mortalidade na alta da UTI		19 (10)	54 (33)	32 (14)	37 (26)

PP-C - posição prona consciente; ONAF - oxigênio nasal de alto fluxo; FiO_2_ - fração inspirada de oxigênio; DSVs - dias sem ventilador, no 28º dia; UTI - unidade de terapia intensiva.

* Inclusive oxigênio nasal de alto fluxo, ventilação não invasiva e invasiva. Os resultados são expressos como mediana (intervalo interquartil 25% - 75%) ou n (%).

Durante a primeira onda, foram intubados 41 (21%) pacientes do Grupo PP-C *versus* 86 (52%) do Grupo Não PP-C, enquanto durante a segunda onda, foram intubados 60 (26%) *versus* 80 (57%) pacientes. No geral, foram intubados 101 (24%) pacientes do Grupo PP-C *versus* 166 (53%) do Grupo Não PP-C. Na população ponderada e ajustada, a RC da IET foi de 0,19 (IC95% 0,10 - 0,28) na primeira onda e de 0,16 (IC95% 0,09 - 0,31) na segunda onda (Figura 2). Na população geral ponderada e ajustada, a RC da IET foi de 0,19 (IC95% 0,10 - 0,28). Não observamos diferenças entre as duas ondas em termos de tamanho do efeito (p = 0,40) (Figura 2). O valor e a análise primária dos efeitos da PP-C na intubação foi de 3,41 na primeira onda e de 4,44 na segunda onda (Figura 7S - Material Suplementar).

**Figura 2:** Risco de intubação e mortalidade intra-hospitalar nos grupos em posição prona em vigília e em posição não prona durante a primeira e a segunda ondas da COVID-19 na Argentina.

RC - razão de chances; IC95% - intervalo de confiança de 95%; PP-C - posição prona consciente. O Grupo Não Posição Prona Consciente foi usado como referência. A população foi ponderada e ajustada por centro, duração da pandemia (semana epidemiológica), estresse do sistema de saúde (ambas as ondas) e taxa de vacinação da população (segunda onda). * I2 = 0,0%, p = 0,401; † I2 = 0,0%, p = 0,440.

Na primeira onda, 19 (10%) pacientes do Grupo PP-C *versus* 54 (33%) do Grupo Não PP-C morreram no hospital, enquanto na segunda onda, morreram 32 (14%) *versus* 37 (26%) pacientes, respectivamente (Tabela 3). Ao todo, morreram 51 (12%) pacientes do Grupo PP-C *versus* 91 (30%) do Grupo Não PP-C. Na população ponderada e ajustada, a RC da mortalidade hospitalar foi de 0,35 (IC95% 0,17 - 0,65) na primeira onda e de 0,22 (IC95% 0,12 - 0,43) na segunda onda (Figura 2). Na população geral ponderada e ajustada, a RC da mortalidade hospitalar foi de 0,25 (IC95% 0,11 - 0,39) e não foram observadas diferenças entre as duas ondas (p = 0,44) (Figura 2).

Os motivos da IET nos 267 pacientes foram a progressão da insuficiência respiratória [n = 259 (97%)] e a insuficiência hemodinâmica [n = 5 (2%)] (Tabela 3). Os efeitos da exposição à PP-C na intubação permaneceram quando foram avaliados conforme a gravidade da insuficiência respiratória definida por PaO_2_/FiO_2_ ≤ 150 durante a primeira e a segunda ondas [RC = 0,25 (0,11 - 0,56) e 0,14 (0,07 - 0,26), respectivamente] (Tabela 3S - Material Suplementar). Os resultados da análise restrita a pacientes com PP-C > 6 horas *versus* pacientes com "zero" horas em posição prona (n = 313) na IET mostraram risco consistentemente reduzido de intubação em ambas as ondas [RC = 0,32 (0,17 - 0,62) e 0,19 (0,10 - 0,33) na primeira e segunda ondas, respectivamente] (Tabela 3S - Material Suplementar).

A tabela 3 apresenta os principais desfechos clínicos. A duração da internação na UTI e no hospital, bem como o número de mortes na UTI, foram menores, e o número de dias sem ventilador (incluindo ONAF) foi maior no Grupo PP-C, em geral e durante a primeira e a segunda ondas.

## DISCUSSÃO

Neste estudo observacional prospectivo e multicêntrico de pacientes com IRA relacionada à COVID-19 que receberam tratamento inicial com ONAF, a PP-C por pelo menos 6 horas por dia foi associada a um menor risco de IET e de mortalidade hospitalar, mesmo após o ajuste de possíveis fatores de confusão. Esses efeitos foram observados em toda a coorte de pacientes e durante cada um dos períodos avaliados, sem diferenças entre a primeira e a segunda onda na Argentina.

A pandemia da COVID-19 levou a uma crise de saúde única na história recente. Durante esse período, as variáveis epidemiológicas, as características dos pacientes infectados, o curso da doença, as intervenções, o prognóstico e os desfechos clínicos dos pacientes mudaram constantemente.^(26)^ Essa situação única e variável torna necessária a avaliação da eficácia dos tratamentos em diferentes contextos.^(27)^ Demonstrou-se que a PP-C é eficaz na redução de IET em pacientes com IRA relacionada à COVID-19 que requerem ONAF quando realizado em UTIs.^(4,28)^ Essas evidências ocorreram principalmente durante a primeira fase da pandemia.^(4,29)^

Em nosso estudo, o tratamento com ONAF e PP-C foi aplicado concomitante e sistematicamente em uma coorte de pacientes consecutivos. Como os grupos de estudo apresentaram diferenças nas variáveis basais, procedeu-se a uma aproximação causal ponderando essas variáveis por meio do método de escore de propensão (IPTW). Os resultados revelaram distribuição equilibrada dos valores das variáveis entre os grupos (em ambas as ondas), permitindo, assim, a suposição de intercambialidade da população.^(18)^ As variáveis incluídas no modelo foram selecionadas pelos DAGs e eram específicas de cada indivíduo ou paciente. No entanto, a eficácia das intervenções ao longo do tempo também pode ser influenciada não apenas por indivíduos, mas também por variáveis contextuais.^(30,31)^ Portanto, após a ponderação por IPTW, ajustamos essas variáveis contextuais. As altas taxas de incidência da COVID-19 e o estresse nos sistemas de saúde foram associados ao aumento da mortalidade.^(6,26,32)^ Para levar em conta essas questões, ajustamos a taxa de incidência da COVID-19 e a proporção de admissão na UTI/admissão hospitalar em cada região do centro participante no momento da admissão do paciente. O SARS-CoV-2 mudou significativamente em termos de sua sequência genética, dando origem a diferentes variantes, que potencialmente podem desempenhar um papel nos resultados clínicos.^(31)^ Na Argentina, a primeira onda foi dominada pela variante selvagem do vírus, enquanto na segunda onda, prevaleceu a variante gama.^(33)^ Por outro lado, as equipes médicas podem ter agido de forma diferente à medida que adquiriram experiência no decorrer da pandemia.^(6,26)^ Para abordar essas questões, ajustamos o período da pandemia em que os pacientes foram incluídos. Os efeitos gerais da vacinação dependem tanto dos efeitos diretos nos indivíduos vacinados quanto dos efeitos indiretos nos indivíduos não vacinados.^(34)^ Para levar em conta esses fatores, incluímos o histórico de vacinação individual e, em seguida, ajustamos a taxa da população vacinada no momento da admissão na UTI em relação à população ponderada. O efeito da exposição (PP-C) nos desfechos clínicos avaliados por inferência causal (IPTW) com ajuste adicional para variáveis contextuais mostrou resultados consistentes em cada uma das ondas. Isso sugere que os efeitos da intervenção são independentes do contexto em que ela é aplicada. É importante ressaltar que a eficácia dos tratamentos não farmacológicos pode sofrer modificações ao longo do tempo, influenciada por mudanças nos fatores contextuais que afetam os desfechos clínicos. Em um estudo de coorte com o objetivo de investigar os resultados de 1.345 pacientes que receberam oxigenação por membrana extracorpórea em decorrência da SDRA devido à COVID-19 durante 4 semestres (2020 - 2021), Schmidt et al. relataram que a mortalidade em 90 dias foi de 42%. A mortalidade foi 10% maior no segundo semestre de 2020 e esteve associada de forma independente à variante delta, mas não a outras variantes.^(35)^

Embora alguns ensaios clínicos tenham demonstrado que a redução do risco de IET em pacientes com IRA relacionada à COVID-19, nenhum estudo demonstrou uma redução no risco de mortalidade.^(3,4)^ No entanto, esses resultados são limitados pelo curto tempo de exposição alcançado em todos os estudos. O tempo de exposição à PP-C parece ser uma questão fundamental para sua eficácia.^(13,28,36)^ A taxa de adesão em nosso estudo foi alta, mais do que o dobro dos tempos de exposição relatados em ensaios clínicos randomizados.^(4)^ Essas taxas poderiam ser explicadas por vários fatores, incluindo as intervenções que estão sendo realizadas em UTIs (onde a observação, o monitoramento e as instruções ao paciente podem ser realizadas de forma mais eficaz) e o uso de opioides de sedação leve ou ambos em mais da metade dos pacientes. Esses momentos poderiam explicar a eficácia, em termos de redução do risco de IET e mortalidade intra-hospitalar alcançada nas duas ondas. Notavelmente, embora as características das populações da primeira e da segunda onda tenham sido diferentes em nosso estudo, a eficácia da intervenção avaliada não foi significativamente diferente em termos de tamanho do efeito.

Nosso estudo tem vários pontos fortes: uma amostra representativa de pacientes com IRA relacionada à COVID-19 de diferentes UTIs com critérios de inclusão consecutivos e tratamento inicial semelhante (ONAF), minimizando o viés de seleção, e tratamento exaustivo de fatores de confusão por meio de inferência causal. As principais limitações deste estudo são aquelas inerentes ao estabelecimento de causalidade por meio de um projeto observacional, ou seja, a possibilidade de não considerar fatores de confusão não medidos. Várias estratégias foram empregadas para minimizar os possíveis vieses inerentes ao desenho: a natureza prospectiva da coorte nos permitiu considerar a maioria dos fatores de confusão conhecidos; o uso do IPTW no ajuste nos permitiu reduzir a multidimensionalidade e equilibrar os fatores que poderiam influenciar a hipótese;^(23)^ além disso, a estimativa do valor eletrônico foi robusta a possíveis fatores de confusão não medidos.^(25)^ No entanto, é necessário enfatizar que os métodos de propensão abordam apenas o viés observado (e não os fatores de confusão não medidos).^(37)^ Além disso, mesmo quando levamos em consideração os fatores de confusão, nunca podemos realmente garantir que capturamos suficientemente todas as informações necessárias, uma vez que as variáveis podem não possuir granularidade adequada.^(37)^ Por fim, outra limitação foi o possível viés derivado da posição não ocultada da equipe de assistência em relação à ordem de intubação. Embora a equipe médica tenha seguido as recomendações predefinidas no protocolo, não registramos o motivo específico da intubação de cada paciente. É importante ressaltar que os resultados da mortalidade por todas as causas, um resultado menos suscetível a vieses, foram consistentes com o desfecho primário (IET), apoiando a hipótese do possível benefício da intervenção. Todos os pacientes incluídos foram tratados em um ambiente de UTI. Portanto, esses resultados não podem ser extrapolados para pacientes assistidos em um ambiente menos complexo ou com doença menos grave.

## CONCLUSÃO

Em pacientes com COVID-19 e insuficiência respiratória aguda admitidos na unidade de terapia intensiva e inicialmente tratados com oxigênio nasal de alto fluxo, a posição prona foi associada à redução no risco de intubação endotraqueal e da mortalidade hospitalar. Esses efeitos foram independentes do contexto em que a intervenção foi aplicada, e não foram observadas diferenças entre as diferentes ondas.
